# Supplementary material for: An open-source framework for large-scale, flexible evaluation of biomedical text mining systems
Source: J Biomed Discov Collab. 2008 Jan 29;3:1. doi: 10.1186/1747-5333-3-1 (PMC2276192; doi:10.1186/1747-5333-3-1)
Supplement: Additional file 1 — Evaluation platform source code. The additional file contains the software evaluation framework discussed in this paper. The most current release can be downloaded from SourceForge.net [1]. See the accompanying README file for instructions on installation and use. Also included in the distribution is a collection of UIMA wrappers for some commonly used BioNLP tools and annotated corpora. [file 1747-5333-3-1-S1.zip › bionlp_uima_repository-1.0.1/docs/api/edu/uchsc/ccp/util/external/kex/KeXEISystem.html]

KeXEISystem (Center for Computational Pharmacology Util/UIMA API)


|  |  |  |
| --- | --- | --- |
| **Overview** \| **Package** \| **Class** \| **Tree** \| **Deprecated** \| **Index** \| **Help** | |  |
| PREV CLASS   NEXT CLASS | **FRAMES**    **NO FRAMES**     **All Classes** |  |
| SUMMARY: NESTED \| FIELD \| CONSTR \| METHOD | DETAIL: FIELD \| CONSTR \| METHOD |  |


---


## edu.uchsc.ccp.util.external.kex Class KeXEISystem

```
java.lang.Object
  edu.uchsc.ccp.util.external.kex.KeXEISystem
```

**All Implemented Interfaces:**: EIFileBasedSystem, EISystem

---

``` public class KeXEISystem extends Object implements EIFileBasedSystem ```

**Author:**
:   williamb
    NOTE: this will only run in a LINUX/UNIX environment due to LINUX specific commands in KeXEISystem.createExecFile

---

**Constructor Summary**

`KeXEISystem(String baseDirectory)`
  
          Initialize a new KeX EI System.


| **Method Summary** | |
| --- | --- |
| `List<TextAnnotation>` | `getEntitiesFromFile(String inputFile)`             Works as it would when using KeX from the command line. |
| `List<TextAnnotation>` | `getEntitiesFromString(String originalText, String originalDocumentID)`             Take a String as input, create a temporary input file, then run KeX just as you would from the command line. |
| `String` | `getOutputFilename()`             Return the outputFileName and path |
| `void` | `reset()`             Deletes the previous output file if it exists. |

**Methods inherited from class java.lang.Object**

`clone, equals, finalize, getClass, hashCode, notify, notifyAll, toString, wait, wait, wait`

**Constructor Detail**

### KeXEISystem

```
public KeXEISystem(String baseDirectory)
```

:   Initialize a new KeX EI System.   
    NOTE: this will only run in a LINUX/UNIX environment due to LINUX specific commands in KeXEISystem.createExecFile

    **Parameters:**: `baseDirectory` - the absolute path to the directory where the bin/ directory is located; there MUST also exist a baseDirectory/work directory


**Method Detail**

### getOutputFilename

```
public String getOutputFilename()
```

:   Return the outputFileName and path

    :   **Specified by:**: `getOutputFilename` in interface `EIFileBasedSystem`
    :   **Returns:**: - String containing the name of the file where the system's output is stored

---


### reset

```
public void reset()
```

:   Deletes the previous output file if it exists. This is necessary because KeX refuses to overwrite an existing output file.

---


### getEntitiesFromFile

```
public List<TextAnnotation> getEntitiesFromFile(String inputFile)
```

:   Works as it would when using KeX from the command line. The input argument is a file where each line represents an individual
    document to be tagged with gene names. Each line must take the form of:  

    :   **Specified by:**: `getEntitiesFromFile` in interface `EIFileBasedSystem`
    :   **Returns:**: - ArrayList of TextAnnotations

---


### getEntitiesFromString

```
public List<TextAnnotation> getEntitiesFromString(String originalText,
                                                  String originalDocumentID)
```

:   Take a String as input, create a temporary input file, then run KeX just as you would from the command line. The document ID is
    appended to the input string in order to create the expected input format.

    :   **Specified by:**: `getEntitiesFromString` in interface `EISystem`


---


|  |  |  |
| --- | --- | --- |
| **Overview** \| **Package** \| **Class** \| **Tree** \| **Deprecated** \| **Index** \| **Help** | |  |
| PREV CLASS   NEXT CLASS | **FRAMES**    **NO FRAMES**     **All Classes** |  |
| SUMMARY: NESTED \| FIELD \| CONSTR \| METHOD | DETAIL: FIELD \| CONSTR \| METHOD |  |


---
